# From Waste to Functional Snack: Drying Kinetics, Neural Network Modeling, and Utilization of Tomato Pomace in Cracker Formulation

**DOI:** 10.1002/fsn3.72008

**Published:** 2026-06-09

**Authors:** Tolga Kağan Tepe, Fadime Begüm Tepe

**Affiliations:** ^1^ Food Technology Department Şebinkarahisar Vocational School, Giresun University Giresun Turkey

**Keywords:** ATR‐FTIR spectroscopy, bioactivity, functional cracker, tomato pomace, valorization

## Abstract

This study investigates the sustainable valorization of tomato pomace (TP), a significant industrial by‐product, by processing it into a tomato pomace powder (TPP) and utilizing it in cracker formulations to support the circular economy. TP was dried using convective oven drying (CO; 50°C, 60°C, 70°C) and microwave (MW; 240, 400 W) methods. The drying kinetics were analyzed using theoretical and semi‐empirical models; the Dincer and Dost model provided the most accurate description of moisture diffusion. The Biot number values, falling within the range of 0.1–100, indicated a mixed regime where both internal and external resistances were present; however, the external convective resistance was identified as the more effective and governing factor. Artificial Neural Network modeling exhibited higher predictive performance compared to the Weibull distribution model under the specific experimental conditions tested in this study. Regarding bioactive retention, a trade‐off was observed: while microwave drying at 400 W maximized the retention of Total Phenolic Compounds and antioxidant activity with the highest energy efficiency, CO drying at 70°C was identified as the optimal method for bakery applications due to superior preservation of carotenoids (lycopene and β‐carotene) and color stability. Crucially, no 5‐Hydroxymethylfurfural was detected in the dried samples. The optimized TPP was incorporated into crackers at 10%, 20%, and 30% levels. This incorporation transformed the crackers into a functional food by introducing significant amounts of lycopene and β‐carotene—compounds absent in the control—while notably increasing Total Phenolic Compounds and antioxidant activity. ATR‐FTIR analysis confirmed physical modification of the matrix and reduced water activity (aw). Although 30% substitution maximized bioactivity, Principal Component Analysis and sensory data identified 10% and 20% substitution levels as the optimum formulations, offering the best balance between functional enrichment and acceptability of sensory acceptability by the panelists.

## Graphical abstract

This study explores a zero‐waste circular economy approach by utilizing industrial tomato pomace as a functional ingredient in cracker production. To prepare the raw material, the drying kinetics of tomato pomace were thoroughly investigated using convective oven drying (50°C–70°C) and microwave drying (240–400 W). The processing behaviors and drying characteristics were systematically evaluated through mathematical modeling, comparing the Weibull model and Artificial Neural Network (ANN) architectures. Following the drying processes, the obtained tomato pomace powder was incorporated into cracker formulations at varying replacement levels (10%, 20%, and 30%). Finally, the developed functional food products were subjected to comprehensive quality characterizations, including bioactive compound analyses, FTIR, and multi‐criteria sensory profiling.

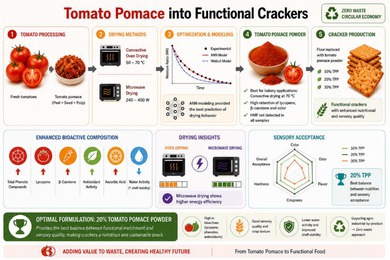

## 1 Introduction

Tomato, which is one of the most cultivated crops in the world, is rich in beneficial health properties and bioactive components such as lycopene, phenolic compounds, and ascorbic acid. Tomato can be consumed as fresh or processed products like juice, paste, puree or ketchup (Lu et al. 2019). According to the Food and Agriculture Organization (FAO), global tomato production was approximately 262.5 million tons. China was the major tomato producer (apx. 70 million tons); India (apx. 20 million tons) and Turkey (apx. 13 million tons) followed China as second and third producers in the world (FAO 2025). One‐third of the total produced tomato is yearly processed, and this process generates huge amounts of wet waste called tomato pomace, which consists of peel, pulp, and seeds. The management of this waste previously included disposal of it in landfills, using it as a natural cheap fertilizer, and using it as animal feed (Chabi et al. 2024). Nowadays, waste management is considered a more important issue, and it is aimed to provide benefits by trying to include waste into the production cycle with the zero‐waste approach, which is a result of the circular economy. Tomato pomace is at least as valuable as tomato due to containing the bioactive and nutritional substances. Being a rich source of dietary fiber (up to 60% of wet dry), carotenoids (especially lycopene and beta carotene), and phenolic compounds make tomato pomace worthful (Belović et al. 2018; Chabi et al. 2024). The high moisture content of tomato pomace causes quick deterioration; therefore, requiring a preservation method. The most useful method is drying.

Drying is a mass and heat transfer process in which water is removed. The main goals of drying are maintaining microbial and chemical safety, minimizing volume, thus providing easier transportation and storage (Kumar et al. 2024). There are various drying methods such as convective drying, microwave drying, freeze drying, etc. The important point to make a choice for drying methods is a higher quality of final product and providing time and energy saving. Convective drying, the most used drying method, has some weaknesses such as longer drying time, higher energy consumption and lower quality characteristics (Llavata et al. 2022). On the other hand, microwave drying has advantages such as higher drying rates, higher energy efficiency and superior product quality (Mouhoubi et al. 2022). Microwave drying, depending on the volumetric heat generated by passing the electromagnetic waves through the food material resulting in molecules' oscillation which produces thermal energy, provides faster moisture evaporation (Jha et al. 2021). Output microwave power is critical in microwave drying due to the fact that although higher output powers provide higher drying rates, they make the quality of final product lack (Keskin et al. 2019). Since the complex cellular matrix of tomato pomace makes it highly sensitive to thermal treatments, accurately identifying the drying behavior through modeling is essential to minimize the loss of heat‐sensitive bioactive compounds.

To understand and identify the drying process, modeling of drying curves is essential. Various mathematical models propose optimizing and designing the drying process (Mouhoubi et al. 2022). Artificial neural networks (ANN) have become a worthwhile approach for modeling complex processes. ANN models, which have strong predictive capability, can accurately predict the drying process depending on input data such as drying temperature, product thickness, drying time, and cabin pressure (Topal et al. 2024). While achieving an optimized powder through advanced modeling is critical, the functional and sensory performance of this powder within a commercial food matrix, such as crackers, must be systematically evaluated to determine its real‐world applicability.

Crackers is a popular and practical snack consumed by people of all ages. Cracker dough generally consists of wheat flour, water, yeast or leavening agent, fats, sugar, and salt. Nutritional value and health beneficial effects of crackers are usually low (Mala et al. 2024). To enhance nutritional value, improve health benefits, and provide cost‐effectiveness, some materials such as fruit and vegetable peel/pulp powders can be incorporated into cracker formulation. There are several research studies in literature investigated by Nakov et al. (2022), Urganci and Isik (2021), and Uppar et al. (2025).

Despite the growing interest in circular economy, specialized research on the drying kinetics of tomato pomace remains relatively scarce in the literature. Existing studies often tend to focus superficially on basic drying parameters or nutrient profiles without exploring complex mass transfer mechanisms. This research addresses this gap by enriching the analysis through advanced diffusion and mathematical modeling coupled with Artificial Neural Networks (ANN). Additionally, the inclusion of energy consumption analysis and ATR‐FTIR spectroscopy establishes a distinctive chemical identity for the resulting powders. Crucially, the utilization potential of tomato pomace powder is demonstrated through its addition to crackers, with findings supported by sensory, nutritional, and spectroscopic data, thereby providing a comprehensive framework for sustainable waste valorization.

Tomato pomace was dried with CO drying at 50°C, 60°C, and 70°C, and microwave drying at 240 and 400 W in the current study. Dried samples were grinded, and tomato pomace powder was obtained. Drying characteristics, energy consumption, bioactive properties (total phenolic compound, antioxidant activity, ascorbic acid, beta carotene, and lycopene), color attributes, and powder properties were investigated. Mathematical and ANN modeling of drying occurred. Additionally, the best powder according to principal component analysis (PCA) was selected and used as a flour substitute in cracker formulation. Bioactive properties, color attributes, and sensory analysis of crackers were evaluated. Besides, some chemical properties of powders and crackers were determined by ATR‐FTIR analyses. The importance of this study is the revalorization of tomato pomace in terms of the zero waste approach. Therefore, the current study can be a solution for the management of waste and demonstrates the potential for new product development.

## 2 Materials and Methods

### 2.1 Material

The tomatoes ( *Lycopersicon esculentum* Rio Grande) were provided from a local grower in the Erbaa district of Tokat, Turkey. At the laboratory, the samples were first washed and the surface was dried. Following that, the tomatoes were processed using a juice extractor (Philips Hr1832/00 Viva Collection, 500 W). The tomato pomace (TP) remaining after the extraction was then collected and used as the main material for this study.

The fresh TP used in the drying experiments was characterized prior to the drying process. The physicochemical properties of the raw material are summarized as follows: The initial moisture content of the fresh samples was determined as 90.64% ± 0.55% (w.b.). The pH, titratable acidity, and total soluble solids (TSS) were found to be 4.69%, 1.37%, and 11.66%, respectively. Additionally, the ash content of the fresh pomace was recorded as 0.59%.

### 2.2 Sample Drying and Powder Production

The TP was dried using two different methods: convective oven (CO) drying and microwave (MW). For all experiments, 100 g of TP was spread in a 2 mm thick layer on the trays to ensure even drying. CO drying was performed at 50°C, 60°C, and 70°C using a static laboratory oven (Nüve FN 400, Turkey) operating under natural convection conditions without active air circulation (forced fan velocity was not applied). The evaporated moisture from the sample matrix was continuously and naturally discharged through the top ventilation ports of the oven. For the microwave method, samples were placed on polypropylene trays and dried at 240 W and 400 W. In both cases, the drying process continued until the samples reached a constant weight. All tests were repeated three times to ensure accuracy. The dried tomato pomace was ground and sieved to a particle size of less than 1000 μm to obtain tomato pomace powder (TPP).

The CO drying temperatures (50°C–70°C) were determined based on preliminary trials and previous research. This range was selected to minimize oxidation risks associated with the high surface area of the mechanically processed pomace at lower temperatures, and to prevent non‐enzymatic browning and aroma degradation at temperatures above 70°C. Additionally, microwave power levels (240 and 400 W) were chosen as the most effective operational range of the equipment to ensure rapid drying without sample surface burning.

### 2.3 Determination of Drying Characteristics

#### 2.3.1 Moisture Content, Moisture Ratio and Drying Rate

Moisture content (Mt) at any given time (g water g^−1^ DM.), moisture ratio (MR), and drying rate (DR) were calculated using standard formulas as described by Demiray et al. (2023).

#### 2.3.2 Drying Characteristics

Effective moisture diffusivity was assessed using the Crank solution, the Anomalous Diffusion model, and the Dincer and Dost method. Following the framework established by Crank (1979), diffusion is categorized based on its relationship with polymer relaxation: Fickian, Case II, or anomalous. While Fick's second law is an approximation, it is widely accepted for analyzing biological products during the falling rate period, providing a reliable average diffusion coefficient. For calculations, Equation (1) was utilized for the infinite slab geometry model, assuming one‐dimensional mass transfer, stable temperature conditions, minimal shrinkage, and a constant diffusion coefficient throughout the process.

(1) $$\mathrm{MR}=\frac{8}{\pi^{2}}\sum_{n=1}^{\infty }\frac{1}{{\left(2n-1\right)}^{2}}\exp\left(-\left(2n-1\right)\pi^{2}\frac{{\text{Deff}}_{1}t}{4L^{2}}\right)$$

Equatio (1) was reduced to Equation (2) by adopting the first‐term approximation for long drying times (Demiray et al. 2023). This simplified relationship was then used to compute the effective moisture diffusivity (Deff_1_), utilizing both the drying duration (*t*) and the specific half‐thickness (*L*) of the tomato pomace.

(2) $$\ln\left(\mathrm{MR}\right)=\ln\left(\frac{8}{\pi^{2}}\right)-\left(\frac{\pi^{2}}{4L^{2}}\;{\text{Deff}}_{1}t\right)$$

By plotting the natural logarithm of MR against the drying time, a clear linear correlation is identified. According to Demiray et al. (2023), the slope derived from this linear plot is expressed through Equation (3) to facilitate further calculations.

(3) $$\text{Slope}=-\frac{\pi^{2}}{4L^{2}}\;{\text{Deff}}_{1}$$

As noted by Simpson et al. (2013), the anomalous diffusion model is particularly effective for analyzing heterogeneous systems. This approach is frequently utilized for food matrices, where structural nonuniformities often create complex and extended pathways for moisture movement.

(4) $$\mathrm{MR}=\frac{8}{\pi^{2}}\sum_{n=1}^{\infty }\frac{1}{{\left(2n-1\right)}^{2}}\mathrm{E\alpha}\left(-\left(2n-1\right)\pi^{2}\frac{{\text{Deff}}_{2}t^{\alpha }}{4L^{2}}\right)$$

In this equation, Eα represents the Mittag‐Leffler function, where α acts as the time exponent (Simpson et al. 2013). It is important to note that when α = 1, this function simplifies to the standard exponential function, aligning the model with Fickian behavior. Furthermore, for extended drying durations, Equation (4) can be reduced to the simplified form of Equation (5).

(5) $$\ln\left(\mathrm{MR}\right)=\ln\left(\frac{8}{\pi^{2}}\right)-\left(\frac{\pi^{2}}{4L^{2}}\;{\text{Deff}}_{2}t^{\alpha }\right)$$

The exponent α acts as the primary indicator for identifying the dominant mass transfer mechanism during the process. Specifically, the drying behavior is classified as sub‐diffusive when 0 < α < 1, while values of α > 1 characterize a super‐diffusive regime. In the specific case where α = 1, the anomalous model aligns perfectly with classical Fickian diffusion, effectively reducing to the Crank model.

Dincer and Dost (1995, 1996) formulated a specialized solution (Equation 6) to Fick's second law, specifically designed for slab geometries.

(6) $$\mathrm{MR}=\sum_{n=1}^{\infty }A_{n}B_{n}\;\text{for}\;0.1\leq \mathrm{Bi}\leq 100\;\text{and}\;\mathrm{Bi}\geq 100$$

Due to the exceptionally small values of the Fourier number (F_0_) in this context, Equation (6) was reduced to the simplified form of Equation (7), as proposed by Bezerra et al. (2015).

(7) $$\mathrm{MR}≅A_{1}B_{1}$$

*A*_1_ and *B*_1_ represent the dimensions of the slab geometry, as given below.

(8) $$A_{1}=G$$

(9) $$B_{1}=\exp\left({-\mu }_{1}^{2}F_{0}\right)$$

The data for moisture ratio versus drying time were analyzed via Equation (10) (Rajoriya et al. 2019) to calculate the moisture diffusivity and mass transfer coefficients.

(10) $$\mathrm{MR}=G\;\exp\left(-\mathrm{St}\right)$$

*G*: The lag factor. *S*: According to Rajoriya et al. (2019), the drying coefficient (s^−1^) represents the drying rate per unit time and is a critical factor in determining the overall drying capacity of the product.

The Biot number was computed by the Equation (11) (Rajoriya et al. 2019).

(11) $$G=\exp\left[\frac{0.2533\mathrm{Bi}}{1.3+\mathrm{Bi}}\right]$$

The moisture diffusivity (*D*, m^2^ s^−1^) was determined by the Equation (12) (Rajoriya et al. 2019).

(12) $$D=\left[\frac{SL^{2}}{\mu_{1}^{2}}\right]$$

μ_1_: characteristic root, If the Bi is between 0.1 and 100, μ_1_ = tan^−1^ (0.640443Bi + 0.380397), If the Bi ≥ 100, μ_1_ = π/2.

The Equation (13) was used for calculation of the coefficient of mass transfer (*h*_m_, m s^−1^) (Rajoriya et al. 2019).

(13) $$h_{m}=\left[\frac{\mathrm{DB}i}{L}\right]$$

#### 2.3.3 Mathematical Modeling

To evaluate the relationship between predicted and experimental MR data, the model with the highest *R* ^2^ and the lowest chi‐square (*χ* ^2^) and Root Mean Square Error (RMSE) values was selected (Tepe and Tepe 2020). The mathematical models evaluated in this study are listed in Table 1. Statistical indicators, namely the fit quality of the models, were evaluated using the *R* ^2^, *χ* ^2^, and RMSE. All nonlinear regression analyses were performed in MATLAB (R2024b) using the curve fitting toolbox with the trust‐region algorithm.

**TABLE 1:** Thin layer models used in the current study.

Model name	Model	References
Weibull distribution	MR = a‐bexp(−kt^n^)	Mandale et al. (2023)
Lewis	MR = exp.(−kt)	Tepe and Tepe (2025)
Henderson and Pabis	MR = aexp(−kt)	Tepe and Tepe (2025)
Page	MR = exp.(−kt^n^)	Tepe and Tepe (2025)
Parabolic	MR = a + bt + ct^2^	Tepe and Tepe (2025)
Midilli and Kücük	MR = aexp(−kt^n^) + bt	Tepe and Tepe (2025)

#### 2.3.4 Artificial Neural Network Modeling

The Artificial Neural Network (ANN) was modeled using the Levenberg–Marquardt backpropagation algorithm within the Neural Network Fitting Toolbox of MATLAB (R2024b, version 8.5). The network architecture was designed with drying time as the input variable and moisture ratio (MR) as the output. A “tansig” transfer function was employed in the hidden layer (Omari et al. 2018), and the optimal number of neurons was determined through an iterative optimization process based on MSE and R^2^ metrics. The experimental dataset was divided into training, test, and validation subsets with a 60:20:20 ratio. The best performance, characterized by the lowest MSE, was achieved using 6 neurons. To ensure generalization and prevent overfitting, training was governed by specific stopping criteria: a maximum of 1000 iterations, six validation checks, and a performance gradient threshold of 1 × 10^−7^ (Yıldız et al. 2015). Finally, model performance was assessed by comparing predictions to experimental data using RMSE and R^2^ values.

#### 2.3.5 Energy Consumption of the Drying Process

Energy consumption was measured using a power meter (Polaxtor, PLX‐15366). The Specific energy consumption (SEC), expressed in kWh kg^−1^ water, was subsequently calculated using Equation (14), following the procedure described by Aksüt et al. (2023).

(14) $$\mathrm{SEC}=\frac{\text{Total consumed Energy}\;\left(\mathrm{kWh}\right)}{\text{Total removed moisture}\;\left(\mathrm{kg}\right)}$$

### 2.4 Determination of Physicochemical Properties

Ash (%), titratable acidity (TA), pH, TSS, and moisture of the TP was determined according to Cemeroğlu (2013). Briefly, moisture was measured gravimetrically in an oven at 105°C while pH and TSS were measured using a digital pH meter and a refractometer, respectively.

### 2.5 Determination of Chemical Properties

#### 2.5.1 Total Phenolic Compound and Antioxidant Activity Analysis

Methanol extraction for total phenolic compound (TPC) and antioxidant activity (AA) was performed according to the procedure described by (Batu and Kadakal 2021). Briefly, 0.5 g of the sample was mixed with 9 mL of a methanol: water solution (90:10) and left overnight. The samples were subsequently centrifuged at 4000 rpm and filtered. The Folin method was conducted using the procedure of Singleton and Rossi (1965), where 300 μL of the extract was mixed with 1500 μL of Folin–Ciocalteu reagent (1:10) and allowed to react for 5 min. Following the addition of 1200 μL of 7.5% sodium bicarbonate, the mixture was incubated in the dark at room temperature for 2 h. Absorbance was measured at 760 nm, and results were expressed as mg gallic acid equivalent (GAE) 100 g^−1^ dry matter (DM). AA was determined via the DPPH method according to the classical procedure of Brand‐Williams et al. (1995). A DPPH solution (in 90% methanol) was prepared to an absorbance of 1.2 at 515 nm. Then, 150 μL of the extract was combined with 2850 μL of the DPPH solution and incubated in the dark for 1 h. Absorbance was recorded at 515 nm, with results reported as mmol Trolox equivalent (TE) 100 g^−1^ DM.

#### 2.5.2 Ascorbic Acid Determination

Ascorbic acid (AscA) content was determined using a modification of the method proposed by Batu and Kadakal (2021). Samples (1 g) were mixed with a 0.5% oxalic acid solution at a 1:9 (w/v) ratio and extracted for 2 h in a dark environment. Following extraction, the samples were centrifuged at 5000 rpm for 10 min, and the resulting supernatant was filtered. The quantitative analysis was based on the methodology of Tabakoglu and Karaca (2018), which relies on the reduction of 2,6‐dichloroindophenol dye by ascorbic acid. The excess dye, which turns pink‐purple under acidic conditions, was measured spectrophotometrically at 518 nm. Ascorbic acid concentrations were calculated using a standard curve and the results were expressed as mg 100 g^−1^ DM.

#### 2.5.3 Lycopene and β‐Carotene Determination

Lycopene and β‐Carotene contents were determined spectrophotometrically following the extraction procedure described by Khamis et al. (2017). Briefly, 0.5 g of dried TPP or 1 g of fresh TP was mixed with a solvent system consisting of hexane, acetone, and ethanol (2:1:1, v/v/v). The mixture was stirred on ice for 15 min, followed by the addition of 3 mL of deionized water and further shaking for 5 min. After allowing 5 min at room temperature for phase separation, the absorbance of the upper hexane layer was measured at 503 nm for lycopene and 453 nm for β‐Carotene using a UV–VIS spectrophotometer. The Equation (15) was used for lycopene content calculation, and a calibration curve obtained at 453 nm was used for β‐Carotene. The results were given as mg 100 g^−1^ DM.

(15) $$\text{Lycopene content}\;\left(\mathrm{mg}\;{\mathrm{kg}}^{-1}\right)=\frac{A_{503}31.2}{g\;\text{sample}}$$

#### 2.5.4 5‐Hydroxymethylfurfural Analysis

The 5‐hydroxymethylfurfural (HMF) content of the samples was determined colorimetrically following the method described by Cemeroğlu (2013) with some modifications. Briefly, 0.5 g of the sample was dissolved in 10 mL of distilled water. Two milliliters of this mixture was transferred into two separate glass tubes, and 5 mL of p‐toluidine solution was added to each. After mixing, 1 mL of distilled water was added to the first tube to serve as a blank, while 1 mL of barbituric acid solution was added to the second tube to initiate the color reaction. The absorbance of the sample was measured against the blank at 550 nm using a spectrophotometer.

### 2.6 Color Analysis

The color properties (L*, a*, b*) of five independent samples were measured using a colorimeter (3NHNR10QC, China). To quantify the color changes, the total color difference (Δ*E*) between the fresh and dried products was calculated via the Equation (16), following the methodology of Horuz et al. (2017a).

(16) $$\Delta E=\sqrt{{\left(L_{0}^{*}-L^{*}\right)}^{2}+{\left(a_{0}^{*}-a^{*}\right)}^{2}+{\left(b_{0}^{*}-b^{*}\right)}^{2}}$$

### 2.7 Powder Properties of Tomato Pomace Powder

#### 2.7.1 Water Holding Capacity

The Water Holding Capacity (WHC) was determined according to the method of Chaudhary and Kumar (2020). Briefly, 1 g of the sample was mixed with 10 mL of pure water, held at room temperature for 30 min, and then centrifuged at 4000 rpm for 30 min.

#### 2.7.2 Bulk and Tapped Bulk Density

Bulk and tapped densities were analyzed using a modified method from Dehghannya et al. (2019). A 5 g sample was placed in a 20 mL measuring cylinder, where bulk density (g cm^−3^) was computed as mass/volume. The tapped density was then determined by recalculating the density after the cylinder had been tapped 20 times on a flat surface.

#### 2.7.3 Carr Index and Hausner Ratio

The flowability of the powder was represented by the Carr Index (CI), while its cohesiveness was indicated by the Hausner Ratio (HR).

Powder properties, including water holding capacity (WHC), Carr Index (CI), and Hausner Ratio (HR), were determined based on the mass‐volume relationships of the bulk and tapped densities as formulated by Chaudhary and Kumar (2020) and Gaikwad et al. (2024).

### 2.8 Cracker Production

Cracker production was carried out according to the method described by Karaman (2016). The samples were prepared based on the formulation presented in Table 2 by incorporating specific proportions of TPP as a replacement for wheat flour. While the control group consisted of 100% wheat flour, the experimental groups involved a progressive reduction in flour content complemented by an increase in TPP. Substitution levels (10%, 20%, and 30%) were determined through preliminary baking trials. A 10% inclusion was selected as the baseline for visible pigmentation. Increments of 10% allowed for systematic evaluation of functional changes. Levels exceeding 30% were excluded because the high fiber and pectin content disrupted dough integrity, prevented proper rising, and caused excessive hardness. During the dough preparation process, flour, TPP, vegetable oil, salt, sugar, and water were blended until a homogeneous consistency was achieved. After shaping, the dough was baked in a preheated oven at 175°C for 15 min. Following the baking process, the crackers were cooled to room temperature and stored under appropriate conditions until further analysis.

**TABLE 2:** Formulation of crackers.

Experiments	Flour (g)	TPP (g)	Oil (g)	Salt (g)	Sugar (g)	Water (g)
Control	50	0	10	1	1	22.5
10% TPP	45	5	10	1	1	22.5
20% TPP	40	10	10	1	1	22.5
30% TPP	35	15	10	1	1	22.5

### 2.9 Sensory Analysis

Sensory evaluation of the crackers was carried out by a panel of 20 semi‐trained individuals. The panelists assessed various attributes, including color, odor, flavor, hardness, crispness, and overall impression, using a 5‐point hedonic scale (ranging from 1: dislike extremely to 5: like extremely) by following the methodology described by Incoronato et al. (2021). The sensory analysis to be conducted within this scope has been deemed ethically acceptable by the Ethics Committee for Research in the Social Sciences, Natural Sciences and Engineering at Giresun University, pursuant to Committee Decision No. 05/300 dated 7 May 2025.

### 2.10 ATR‐FTIR Analysis of Powders and Crackers

Fourier transform infrared spectroscopy (FTIR) was employed to characterize the chemical spectra of the powder and cracker samples. Measurements were conducted using a VERTEX 70 Series FTIR spectrometer (Bruker, Germany) equipped with an ATR cell. Fresh samples were analyzed as 0.5‐cm sections, whereas dried samples were ground and placed onto a multiple‐bounce ZnSe crystal. Scans were performed at a resolution of 2 cm^−1^ over a spectral range of 400 to 4000 cm^−1^. The resulting spectral data were baseline‐corrected using a zero reference and normalized against the maximum absorbance value. All data transformations were executed using Spectragryph (version 1.2.14) software.

### 2.11 Statistical Analysis

Statistical analysis was conducted using SPSS 22.0 software (IBM Corporation, Armonk, NY). Experimental results are expressed as the mean ± standard deviation (SD). Analysis of variance (ANOVA) was employed to determine significant differences at a significance level of *p* = 0.05, and specific differences between group means were identified using Duncan's post hoc test. Additionally, Principal Component Analysis (PCA) was performed using Minitab 21 software to evaluate the relationships between variables.

## 3 Results and Discussion

### 3.1 Drying Characteristics and Energy Consumption

Drying parameters were described with specific parameters detailed in Table 3. All samples reached a final moisture content of ~0.05 g water g^−1^ DM. The resulting a_w_ values (0.42–0.43) remained well within the microbiological safety region (a_w_ < 0.60), consistent with the safety standards recommended by Alp and Bulantekin (2021) and Karuppuchamy et al. (2024). In the current study, the dried TP samples were under the microbiological safety region in terms of a_w_.

**TABLE 3:** Drying parameters and water activities of hot air and microwave dried tomato pomace.

Experiments	G	Bi	u1	S (s^−1^)	D (m^2^ s^−1^)	Deff_1_ (m^2^ s^−1^)	Deff_2_ (m^2^ s^−1^)	α	hm (m s^−1^)	a_w_ ± SD
CO‐50°C	1.1071	0.76884	0.71740	5.10 × 10^−5^	3.97 × 10^−10^	5.59 × 10^−11^	8.85 × 10^−11^	0.94249	1.52 × 10^−7^	0.43 ± 0.001^a^
CO‐60°C	1.0968	0.68868	0.68763	7.43 × 10^−5^	6.28 × 10^−10^	8.09 × 10^−11^	1.04 × 10^−10^	0.96335	2.16 × 10^−7^	0.42 ± 0.005^a^
CO‐70°C	1.1009	0.7207	0.69981	1.01 × 10^−4^	8.22 × 10^−10^	1.05 × 10^−10^	1.26 × 10^−10^	0.9796	2.96 × 10^−7^	0.43 ± 0.003^a^
MW 240 W	1.1849	1.4753	0.92435	7.60 × 10^−4^	3.56 × 10^−9^	7.94 × 10^−10^	1.87 × 10^−10^	1.1992	2.63 × 10^−6^	0.42 ± 0.001^a^
MW 400 W	1.1869	1.4967	0.92928	1.57 × 10^−3^	7.26 × 10^−9^	1.66 × 10^−9^	4.44 × 10^−10^	1.200	5.43 × 10^−6^	0.43 ± 0.002^a^

*Note:* Different letters in the same column indicate significant differences with confidence of 95%.

Abbreviations: CO, convective oven dried samples; MW, microwave dried samples; SD, standard deviation.

To elucidate moisture transport, three mathematical frameworks were utilized: Crank, Anomalous, and Dincer & Dost. Drying times decreased significantly with increasing air temperature and microwave power, from 810 min (50°C) to 390 min (70°C) and 46 min (240 W) to 23 min (400 W). These findings align with Ibrahim et al. (2017) for tomato pomace and Mennouche et al. (2023) for tomato waste dried by CO drying method. As reported by Beigi (2016), increased drying temperatures enhance the heat transfer rate between the food and air, leading to higher evaporation rates that shorten drying time. Similarly, Tepe and Tepe (2020) attributed the reduced drying times in microwave processes to the greater heating of water molecules at higher power levels.

The observed increases in drying capacity (S), mass transfer coefficient (h_m_), and effective diffusivity (D, D_eff1_, D_eff2_) (Table 3) support these arguments. Increasing temperature facilitated thermal energy accessibility, thereby increasing S and h_m_ as a result of the stimulation of water molecules, a trend consistent with findings for tomato waste, blueberry pulp and apple slices (Mennouche et al. 2023; Rurush et al. 2022; Rajoriya et al. 2019). Similar trends were reported for microwave‐dried potato and kiwi fruit slices (Tepe 2025; Darvishi et al. 2018).

**FIGURE 1:**
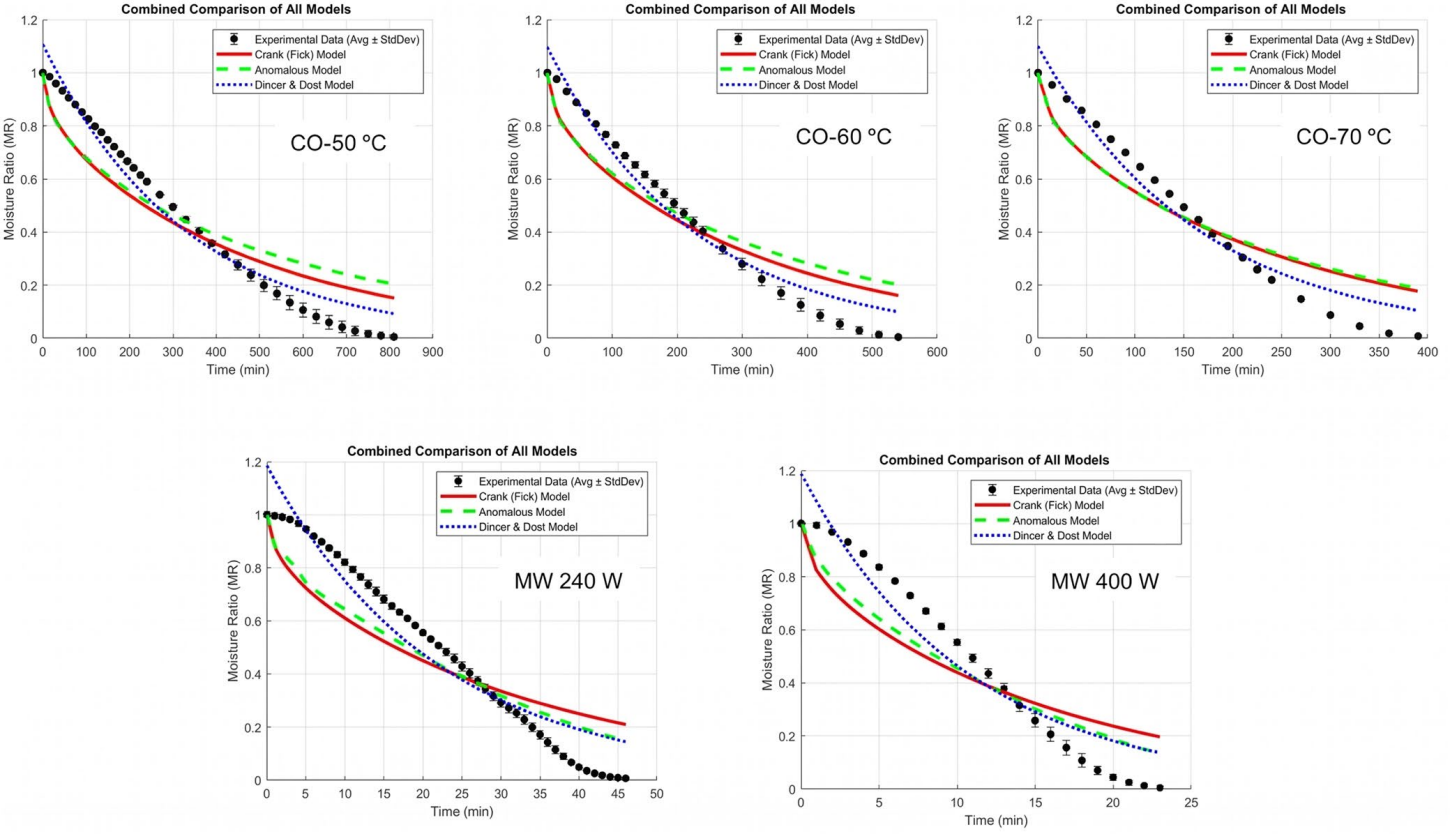
Diffusion models of samples dried by CO and MW drying methods.

The Dincer and Dost approach provided the highest predictive accuracy for TP diffusion (higher R^2^ and lower RMSE, Figure 1 and Table 4). Calculated Biot numbers consistently fell near the lower limit of the mixed regime (Bi ~0.1), suggesting that external convective resistance might be the primary barrier to moisture transfer, potentially overshadowing internal resistance. Similar dominance of surface resistance has been noted by Górnicki et al. (2019), Tepe (2025), and Dursun Saydam (2025). Specific energy consumption (SEC) varied significantly (*p* < 0.05) (Table 4). In CO drying, increasing temperature from 50°C to 60°C significantly reduced SEC, while MW drying demonstrated superior energy efficiency, yielding SEC values approximately four times lower than CO. This is attributed to the volumetric heating effect, with 400 W identified as the energy‐efficient condition for this application.

**TABLE 4:** Statistical data and specific energy consumption of different diffusion approaches.

Experiments	Crank	Anomalous	Dincer and Dost	SEC (kWh kg^−1^ water) ± SD
CO‐50°C	*R* ^2^ = 0.8662 RMSE = 0.12186	*R* ^2^ = 0.8274 RMSE = 0.13839	*R* ^2^ = 0.9689 RMSE = 0.05868	9.01 ± 0.019^a^
CO‐60°C	*R* ^2^ = 0.8692 RMSE = 0.11460	*R* ^2^ = 0.8410 RMSE = 0.12641	*R* ^2^ = 0.9673 RMSE = 0.05728	8.28 ± 0.071^b^
CO‐70°C	*R* ^2^ = 0.8585 RMSE = 0.11753	*R* ^2^ = 0.8479 RMSE = 0.12189	*R* ^2^ = 0.96123 RMSE = 0.06152	8.24 ± 0.320^b^
MW 240 W	*R* ^2^ = 0.7794 RMSE = 0.15588	*R* ^2^ = 0.8559 RMSE = 0.12599	*R* ^2^ = 0.92682 RMSE = 0.089782	2.41 ± 0.003^c^
MW 400 W	*R* ^2^ = 0.7751 RMSE = 0.16478	*R* ^2^ = 0.8587 RMSE = 0.1306	*R* ^2^ = 0.9182 RMSE = 0.099369	2.06 ± 0.004^c^

*Note:* Different letters in the same column indicate significant differences with confidence of 95%.

Abbreviations: CO, convective oven dried samples; MW, microwave dried samples; SD, standard deviation; SEC, specific energy consumption.

### 3.2 Modeling of Tomato Pomace Drying

**FIGURE 2:**
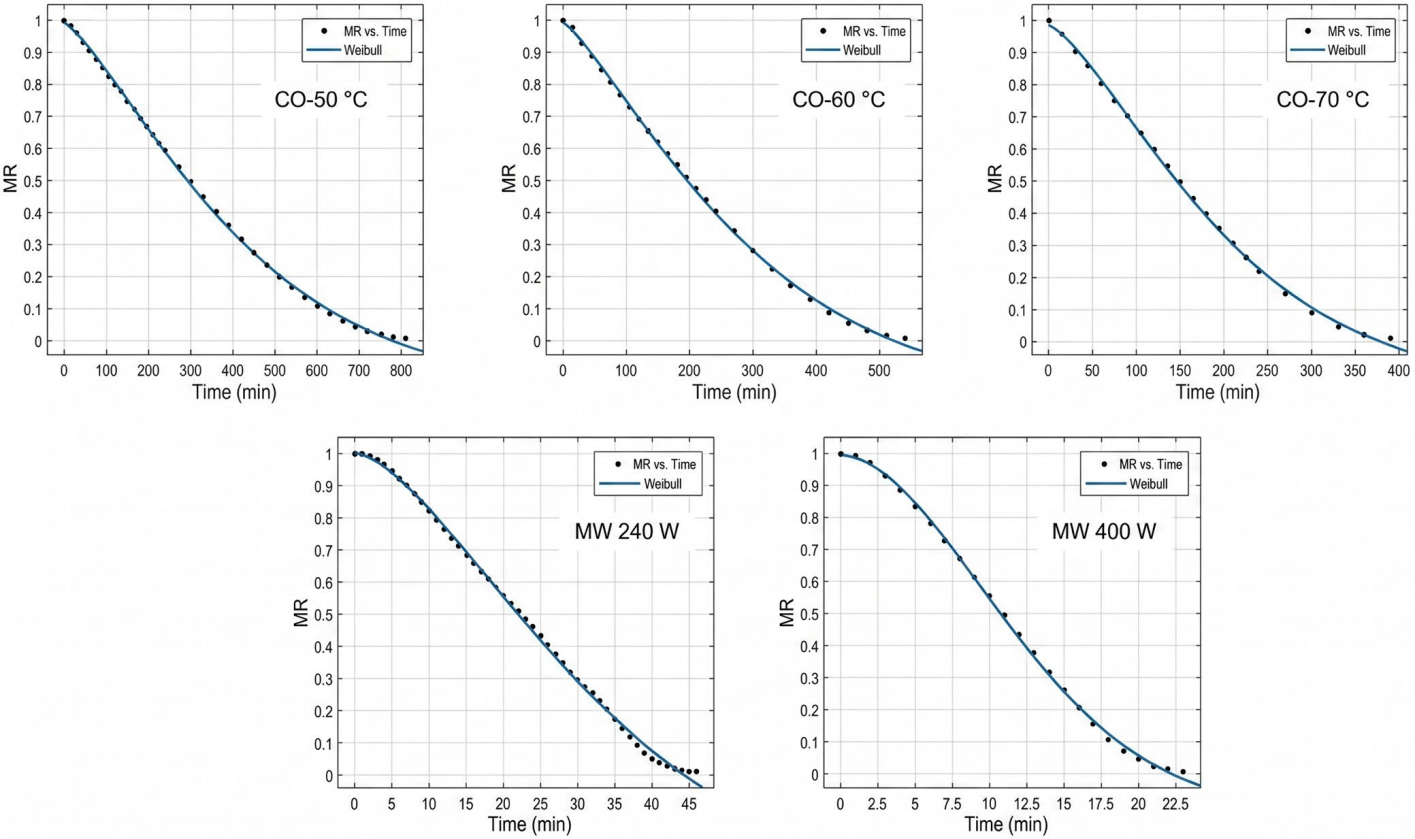
Weibull distribution curves for CO and MW dried samples.

**FIGURE 3:**
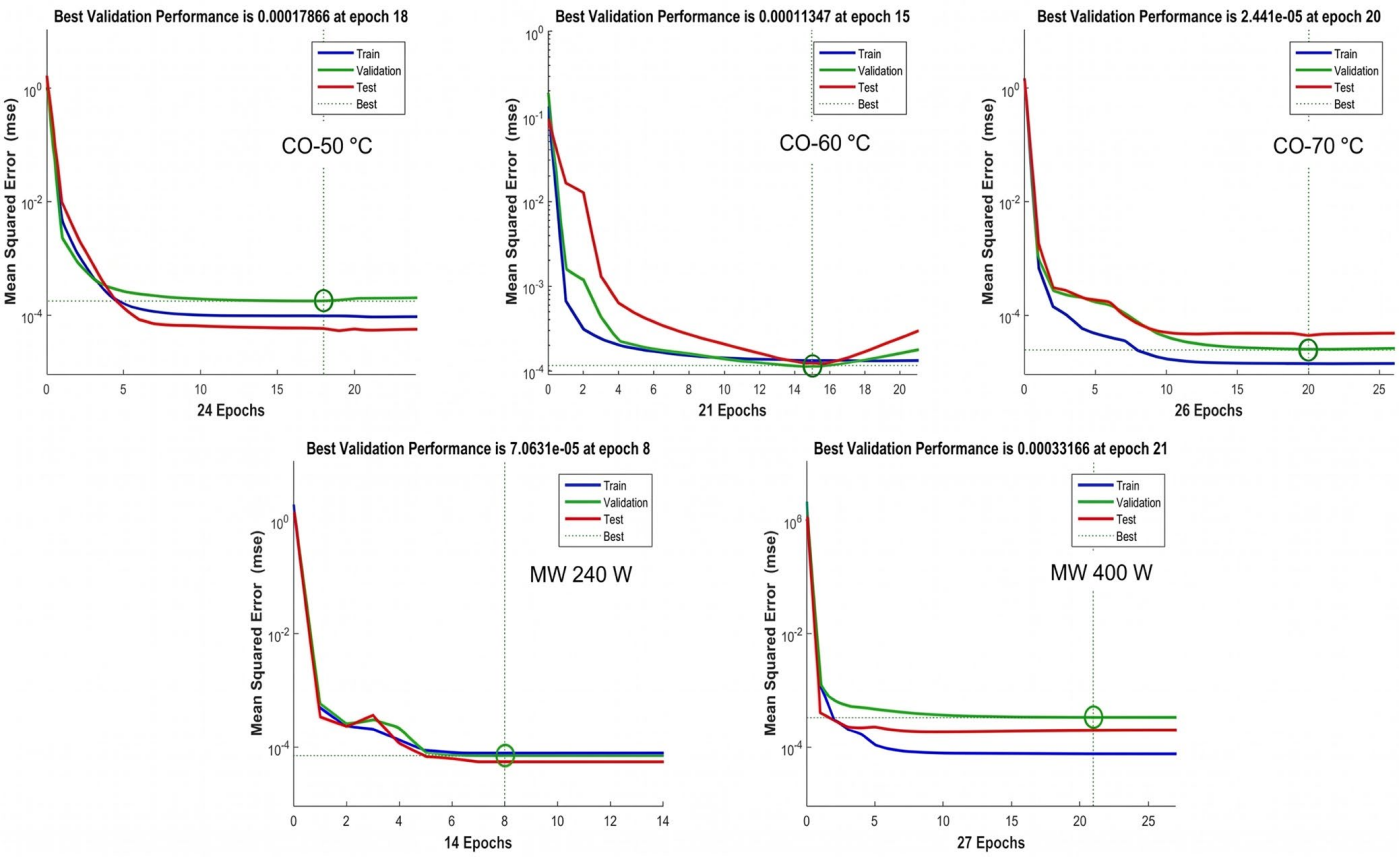
Best validation performance of ANN models.

**FIGURE 4:**
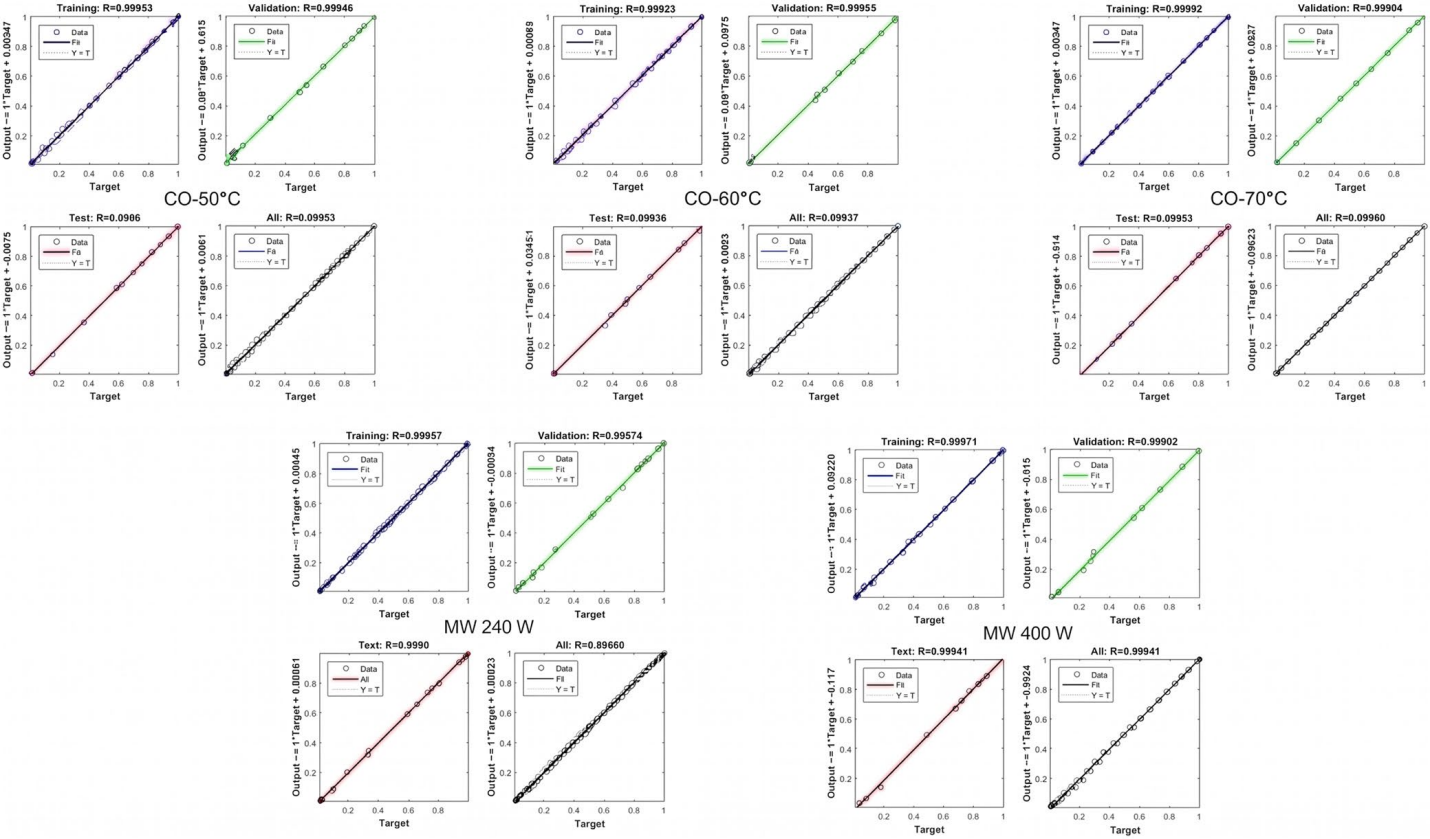
Regressions of ANN modeling.

**FIGURE 5:**
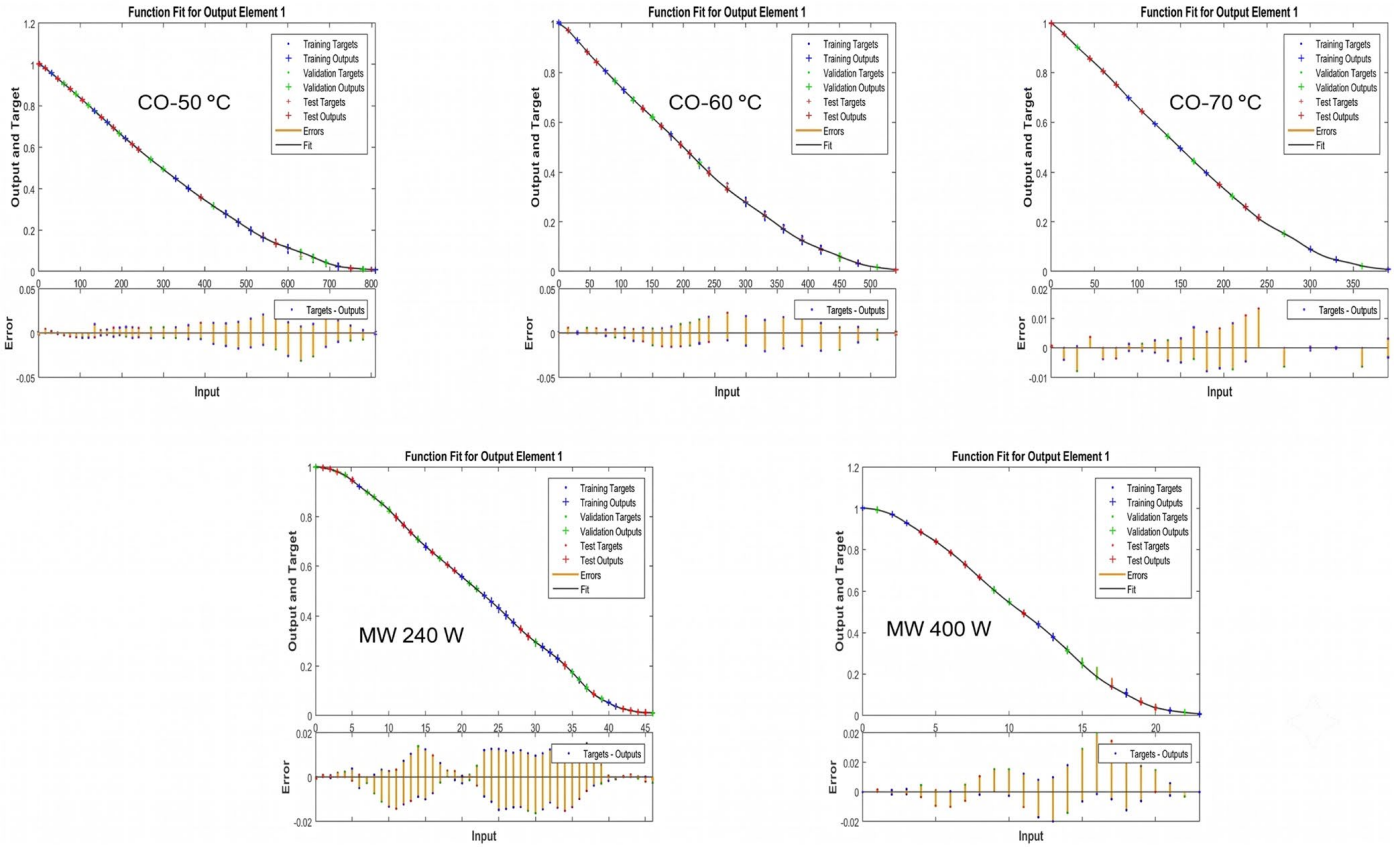
Fitting of ANN modeling.

The moisture ratio data fitted to six thin‐layer models, with the Midilli et al. and Weibull distribution model consistently providing the most accurate predictions (*R* ^2^ > 0.9987, Table 5; Figure 2). Mennouche et al. (2023) and Al‐Harahsheh et al. (2009) reported that Proposed and Midilli et al. models described tomato waste drying curves for CO and MW methods at their empirical conditions. Their low *χ* ^2^ and RMSE values indicate a superior capacity to characterize TP drying kinetics compared to other semi‐empirical models at tested conditions. Beyond traditional regression, an ANN topology was developed (Figures 3, 4, 5), which exhibited even higher predictive accuracy and lower error rates. A critical challenge in ANN applications is overfitting, typically identified by diverging test and validation trends (Kurtulmuş et al. 2020); however, the identical trends observed in this study (Figure 3) confirm effective model generalization.

**TABLE 5:** Statistical data and model constants of thin layer modeling.

Models	Experiments	Model constants				*χ* ^2^	RMSE	*R* ^2^
Page	CO‐50°C	*k* = 0.0001726	*n* = 1.470			0.000551385	0.02282	0.9956
	CO‐60°C	*k* = 0.0003323	*n* = 1.456			0.00063983	0.02434	0.9945
	CO‐70°C	*k* = 0.0003589	*n* = 1.524			0.000628857	0.02391	0.9947
	MW 240 W	*k* = 0.001885	*n* = 1.927			0.000960794	0.03033	0.992
	MW 400 W	*k* = 0.005256	*n* = 2.068			0.000540068	0.02225	0.9962
Henderson and Pabis	CO‐50°C	*k* = 0.003062	*a* = 1.107			0.003838494	0.06021	0.9691
	CO‐60°C	*k* = 0.004453	*a* = 1.097			0.003802934	0.05934	0.9675
	CO‐70°C	*k* = 0.006033	*a* = 1.101			0.004572019	0.06447	0.9613
	MW 240 W	*k* = 0.04564	*a* = 1.185			0.008765387	0.09161	0.9271
	MW 400 W	*k* = 0.09398	*a* = 1.187			0.011708684	0.1036	0.9185
Midilli and Kucuk	CO‐50°C	*k* = 0.0002965	*a* = 0.9888	*n* = 1.345	*b* = −0.0001303	**8.27605E‐05**	**0.008577**	**0.9994**
	CO‐60°C	*k* = 0.0006118	*a* = 0.9894	*n* = 1.306	*b* = −0.0002212	**0.000106459**	**0.009523**	**0.9992**
	CO‐70°C	*k* = 0.0004842	*a* = 0.9814	*n* = 1.431	*b* = −0.0002482	**0.000143353**	**0.01083**	**0.999**
	MW 240 W	*k* = 0.003429	*a* = 1.007	*n* = 1.625	*b* = −0.004528	**0.000169421**	**0.01245**	**0.9987**
	MW 400 W	*k* = 0.006438	*a* = 0.9955	*n* = 1.915	*b* = −0.003965	**0.00013407**	**0.01057**	**0.9992**
Weibull Distribution	CO‐50°C	*k* = 0.0003371	*a* = −0.1503	*n* = 1.307	*b* = 1.140	**7.41031E‐05**	**0.008116**	**0.9995**
	CO‐60°C	*k* = 0.0006815	*a* = −0.1781	*n* = 1.267	*b* = 1.169	**9.56371E‐05**	**0.009026**	**0.9993**
	CO‐70°C	*k* = 0.0005545	*a* = −0.1349	*n* = 1.390	*b* = 1.117	**0.000134237**	**0.01048**	**0.9991**
	MW 240 W	*k* = 0.004241	*a* = −0.3547	*n* = 1.520	*b* = 1.359	**0.000165902**	**0.01232**	**0.9987**
	MW 400 W	*k* = 0.007313	*a* = −0.1187	*n* = 1.847	*b* = 1.112	**0.000134324**	**0.01058**	**0.9992**
Lewis	CO‐50°C	*k* = 0.002711				0.005568703	0.07358	0.9525
	CO‐60°C	*k* = 0.003988				0.005057977	0.06979	0.9533
	CO‐70°C	*k* = 0.005406				0.00579739	0.07439	0.9459
	MW 240 W	*k* = 0.03801				0.013139116	0.1134	0.8857
	MW 400 W	*k* = 0.07868				0.01609632	0.1242	0.8775
Parabolic	CO‐50°C	*a* = 1.032	*b* = −0.002125	*c* = 0.000001017		0.000171274	0.01253	0.9987
	CO‐60°C	*a* = 1.026	*b* = −0.003122	*c* = 0.000002196		0.000145437	0.01137	0.9989
	CO‐70°C	*a* = 1.033	*b* = −0.004259	*c* = 0.000003955		0.000381857	0.01816	0.9971
	MW 240 W	*a* = 1.064	*b* = −0.02669	*c* = 0.00004966		0.000556746	0.02283	0.9956
	MW 400 W	*a* = 1.080	*b* = −0.05778	*c* = 0.0003434		0.001597728	0.03739	0.9899
ANN	CO‐50°C						**0.010271271**	**0.9995**
	CO‐60°C						**0.011256376**	**0.9994**
	CO‐70°C						**0.004720434**	**0.9999**
	MW 240 W						**0.008466788**	**0.9997**
	MW 400 W						**0.012432216**	**0.9994**

*Note:* Bold indicates the most appropriate models.

Abbreviations: CO, convective oven dried samples; MW, microwave dried samples.

As highlighted by Onwude et al. (2016), the ANN approach offers a wider operational range and more robust performance under varying experimental conditions compared to mathematical frameworks. Our findings align with recent literature where ANN outperformed conventional methods for diverse materials and techniques, including CO‐dried *Moringa oleifera* (Thuy et al. 2025), potato slices (Chokphoemphun et al. 2024; Tepe 2025), and Refractance Window‐dried onion puree and coriander (Zalpouri et al. 2023, 2025). This suggests that ANN provides a highly reliable and flexible tool for characterizing drying behavior across the specific range of conditions applied in this study.

### 3.3 Powder Properties of Tomato Pomace Powders

Water holding capacity (WHC), Carr Index (CI) and Hausner Ratio (HR) of TPP were given in Table 6. The water holding capacity of a power is a hydration parameter that assesses the powder's ability to absorb water and attain the correct consistency (Alam et al. 2023). WHC of dried tomato powders ranged from 848.33% to 1105.56%. The highest WHC was obtained from CO dried samples at 50°C, while the lowest one was MW dried samples at 240 W. The higher WHC, the lower temperature in HA drying. On the other hand, increasing microwave power slightly increased WHC. Likewise, İzli et al. (2022) reported that increasing drying temperature caused decrement in WHC, while increasing microwave power increased WHC of pumpkin samples. WHC is a function of hydrophilic molecules such as carbohydrates and proteins, thus has a positive correlation with amounts of these molecules (Abe‐Inge et al. 2018). Lower drying temperatures may cause more retention of proteins and carbohydrates. Therefore, the increment in WHC at lower temperatures can be explained with this phenomenon. The flowability and cohesiveness of powder products were described by Carr Index (CI) and Hausner Ratio (HR), respectively. CI and HR of TPP ranged from 21.63 (60°C CO) to 14.26 (240 W MW) and from 1.28 (60°C HA) and 1.17 (240 W MW). The higher CI, the lower flowability. Additionally, lower cohesion properties (lower HR) are related to higher flowability. According to Aziz et al. (2018) flowability of powders identified as “good” when CI values ranged from 11 to 15. If CI values varied from 16 to 20, flowability of powders is classified as “fair.” CI values between 21 and 25 indicate “passable” flowability properties (Aziz et al. 2018). It was observed that there is no statistically important effect of different drying temperatures on CI values. However, microwave drying technique dramatically increased the flowability properties of TPP. Increasing microwave power caused a decrement in flowability. Similarly, İzli et al. (2022) noted that microwave drying resulted more flowable pumpkin powder than CO. The shorter drying time caused by microwave drying may have limited the interaction between particles in the TP matrix. Thus, a product with more flowable powder properties may have been obtained at the end of the microwave drying process.

**TABLE 6:** Powder properties and color attributes of tomato pomace powders.

Experiments	WHC (%) ± SD	CI (%) ± SD	HR ± SD	L* ± SD	a* ± SD	b* ± SD	ΔE
Fresh	—	—	—	38.30 ± 0.25^c^	19.35 ± 0.90^c^	11.44 ± 0.76^c^	0
CO‐50°C	1105.56 ± 5.56^a^	21.50 ± 0.45^a^	1.27 ± 0.007^a^	57.85 ± 0.50^b^	24.50 ± 0.33^a^	17.82 ± 0.40^b^	21.20
CO‐60°C	1055.56 ± 100^ab^	21.63 ± 1.63^a^	1.28 ± 0.027^a^	57.91 ± 0.40^b^	25.90 ± 1.32^a^	18.33 ± 0.42^b^	21.80
CO‐70°C	972.50 ± 7.5^b^	20.14 ± 0.24^ab^	1.25 ± 0.004^ab^	59.39 ± 0.71^a^	25.40 ± 0.27^a^	20.31 ± 0.34^a^	23.55
MW 240 W	848.33 ± 18.33^c^	14.26 ± 0.74^c^	1.17 ± 0.010^c^	59.36 ± 0.39^a^	24.97 ± 1.11^a^	20.60 ± 0.52^a^	24.05
MW 400 W	943.33 ± 76.67^cd^	18.06 ± 1.94^b^	1.22 ± 0.029^b^	56.98 ± 1.39^b^	21.60 ± 0.43^b^	19.95 ± 0.40^a^	20.65

*Note:* Different letters in the same column indicate significant differences with confidence of 95%.

Abbreviations: CI, Carr Index; CO, convective oven dried samples; HR, Hausner ratio, L*, a*, b* and Δ*E* color parameters; MW, microwave dried samples; SD, standard deviation; WHC, water holding capacity.

### 3.4 Color Properties of Tomato Pomace Powder

The color characteristics of fresh TP and the TPP obtained after drying and grinding are presented in Table 6. The color values of the dried powders differed significantly from the fresh sample (*p* < 0.05). The lightness (L*) value of the fresh pomace was measured as 38.30. Following the drying and subsequent grinding processes, a significant increase in L* values was observed in all powder samples, ranging from 56.98 to 59.39. This substantial increase in brightness may be primarily attributed to the reduction in particle size, which enhances light scattering. According to Li et al. (2020), the optical properties of powders depend heavily on light scattering; as the scattering phenomenon becomes more pronounced with finer particles, the powder color appears brighter. Furthermore, chemical changes induced by thermal processing also contribute to this lightning. Thermal treatment triggers a reversible isomerization of trans‐lycopene to cis‐isomers. These cis‐isomers are chemically more unstable and highly susceptible to oxidation. Consequently, the oxidation of these pigments leads to color bleaching (Castoldi et al. 2015). Also, the increase in L* values is also attributed to the lightening of the color resulting from oxidation (Lv et al. 2020). Redness (a*) increased in all dried samples compared to the fresh control (19.35) due to the concentration of pigments after moisture removal. The highest redness values were observed in CO‐60°C (25.90) and CO‐70°C (25.40). However, the redness value for the MW 400 W treatment (21.60) was significantly lower than other conditions, suggesting that high microwave power may have caused thermal degradation of heat‐sensitive pigments. In fresh tomatoes, lycopene predominantly exists in the thermodynamically most stable all‐trans form. However, thermal processing triggers the isomerization of the trans‐form to the cis‐form. Although the cis‐form is chemically unstable and susceptible to degradation, it exhibits relatively higher solubility in organic solvents and greater bioavailability compared to the trans‐form (Bhatkar et al. 2021). In the present study, although analytical results indicated a reduction in lycopene content on a dry matter basis due to thermal degradation and oxidation of the unstable cis‐isomers, a significant increase in a* values was observed in the dried powders compared to the fresh control. This apparent contradiction is attributed to the physical concentration effect. Since fresh TP has a very high moisture content, the removal of water concentrates the remaining pigments per unit mass. Furthermore, the thermal disruption of the cellular matrix likely enhanced the release and surface visibility of the pigments, resulting in higher chromatic values despite the chemical loss of lycopene. Regarding the yellowness (b*) values, a consistent upward trend was observed in all dried samples. While the fresh TP had the lowest b* value of 11.44, this value significantly increased to a range of 17.82–20.60 in the dried powders. The highest yellowness values were recorded in the CO‐70°C (20.31) and MW 240 W (20.60) samples, which were statistically categorized in the same group. Similar to lycopene, β‐carotene is susceptible to degradation via isomerization and oxidation during thermal processing, leading to a reduction in its chemical content (Bhatkar et al. 2021). However, the b* values, representing yellowness, exhibited a significant increase in all dried samples compared to the fresh control (*p* < 0.05). This inverse relationship between quantitative chemical loss and the enhanced colorimetric value is primarily driven by the physical concentration of pigments. The removal of moisture results in a denser packing of the remaining carotenoids per unit mass, thereby intensifying the visible yellowness despite the loss of bioactive compounds. The total color difference (Δ*E*) values varied between 20.65 and 24.05, indicating a distinct visual difference between the fresh material and the final powder products.

### 3.5 Total Phenolic Compound, Antioxidant Activity and Ascorbic Acid Content of Tomato Pomace Powders

Total phenolic compounds (TPC), antioxidant activity (AA), and ascorbic acid (AscA) contents of fresh and dried samples were given in Table 7. TPC, AA, and AscA of fresh samples were found to be 399.54 mg GAE 100 g^−1^ DM, 1.88 mmol TE 100 g^−1^ DM, and 274.86 mg 100 g^−1^ DM, respectively. TPC of dried samples reduced with drying and ranged from 154.21 to 427.81 mg GAE 100 g^−1^ DM. Increasing drying temperature and microwave power provided less loss of TPC. There was no statistically significant difference between fresh and MW dried at 400 W power level samples. The highest loss of TPC was observed in CO dried samples at 50°C. AA of TP reduced after drying for all parameters. AA of dried samples ranged from 0.76 to 1.63 mmol TE 100 g^−1^ DM. The highest loss of AA was observed in CO dried samples at 50°C, while MW drying at 400 W power level caused the minimum loss of AA. Increasing drying temperature and microwave power procured higher retention of AA. Petković et al. (2025) determined higher TPC and AA in tomato waste dried at 70°C than those at 50°C. Similarly, in other studies, Kaur et al. (2020) and Chikpah et al. (2022) noted that increasing drying temperatures provided increasing TPC and AA retention of tomato, sweet pepper, and pumpkin slices. Higher retention of TPC and AA at higher temperatures may be related to the increment in the release of bound phenolics from cell walls because of breaking down the esters which is caused by heat treatment. Additionally, longer drying times required by lower drying temperatures can cause a decrement in TPC and AA due to oxidative stress. Likewise, İzli et al. (2022) indicated that increasing drying temperature in CO and increasing microwave power in microwave drying caused an increment in TPC. Higher retention of TPC and AA in microwave dried samples can be in to the releasing of more bound phenolic substances by breaking down the cell wall in microwave drying or the formation of new phenolic substances resulting from the Maillard reaction.

**TABLE 7:** Total phenolic compound, antioxidant activity, ascorbic acid, HMF, lycopene, and β‐carotene content of fresh tomato pomace and powders.

Experiments	TPC (mg GAE 100 g^−1^ DM) ± SD	AA (mmol TE 100 g^−1^ DM) ± SD	AscA (mg 100 g^−1^ DM) ± SD	HMF ± SD	Lycopene (mg 100 g^−1^ DM) ± SD	β‐Carotene (mg 100 g^−1^ DM) ± SD
Fresh	399.54 **±** 48.19^a^	1.88 ± 0.32^a^	274.86 ± 54.25^a^	—	909.51 ± 129.29^a^	83.54 ± 11.78^a^
CO‐50°C	154.21 **±** 6.88^c^	0.76 ± 0.075^d^	19.74 ± 11.02^d^	ND	531.22 ± 56.70^b^	49.51 ± 5.60^b^
CO‐60°C	174.16 **±** 12.56^c^	0.84 ± 0.11^d^	68.84 ± 6.28^cd^	ND	523.13 ± 47.93^b^	48.32 ± 4.46^b^
CO‐70°C	203.87 **±** 5.65^bc^	1.10 ± 0.13^cd^	99.12 ± 13.72^bc^	ND	559.86 ± 52.73^b^	51.16 ± 1.92^b^
MW 240 W	274.06 **±** 17.59^b^	1.42 ± 0.07^bc^	143.75 ± 35.19^b^	ND	460.29 ± 73.15^b^	44.40 ± 7.19^b^
MW 400 W	427.81 **±** 56.48^a^	1.63 ± 0.12^ab^	100.93 ± 28.56^bc^	ND	432.83 ± 45.64^b^	46.35 ± 4.62^b^

*Note:* Different letters in the same column indicate significant differences with confidence of 95%.

Abbreviations: AA, antioxidant activity; AscA, ascorbic acid content; CO, convective oven dried samples; MW, microwave dried samples; ND, not detected; SD, standard deviation; TPC, total phenolic compound.

The content of AscA significantly decreased with drying. The highest loss of AscA was observed in CO dried samples at 50°C (19.74 mg 100 g^−1^ DM), while MW drying at 240 W power level provided the highest retention of AscA (143.75 mg 100 g^−1^ DM). HA drying of TP caused higher loss of AscA compared to microwave drying. Similarly, Alibas and Yilmaz (2022) reported that microwave drying of orange slices provided more retention of AscA compared to CO drying. Increasing drying temperature caused lower loss of AscA due to lower drying time and thus lower exposition to oxygen and heat treatment. Zia and Alibas (2021) stated that increasing drying temperature and microwave power raised AscA content of cornelian cherries. Wei et al. (2022) notified that increasing microwave power up to 280 W increased ascorbic acid retention, while higher microwave powers above 280 W increased AscA loss. When the applied microwave power was excessively high, the intense friction and interactions among polar molecules likely led to greater heat buildup in the core of the sample. This localized overheating may have accelerated the degradation of AscA, which is both acidic and highly sensitive to heat, thereby lowering its retention. Conversely, when the microwave power was too low, prolonged heating at relatively low temperatures also contributed to increased AscA loss (Wei et al. 2022). Alibas and Yilmaz reported that microwave drying at 350 W microwave power level provided the best protection of AscA, while too high and too low microwave powers adversely affected AscA. Additionally, it was noted that lower drying temperatures can cause oxidation due to longer drying time, thus can cause loss of AscA, and higher drying temperatures increased loss of AscA as heat sensitivity (Alibas and Yilmaz 2022).

### 3.6 Lycopene and β‐Carotene Content of Tomato Pomace Powders

The lycopene and β‐Carotene content of fresh TPand TPP are given in Table 7. The lycopene content of the fresh TP was determined as 909.51 mg 100 g^−1^ DM. Following the drying operations, a statistically significant decrease was observed in the lycopene content of all powder samples compared to the fresh control (*p* < 0.05). The lycopene values of the dried powders ranged between 432.83 and 559.86 mg 100 g^−1^ DM. According to the statistical analysis, all drying treatments—regardless of the method (CO or MW) or process parameters (temperature or power)—showed no mean differences. This indicates that while drying caused a reduction compared to fresh material, no significant differences in lycopene retention were observed among the different drying conditions applied. Similar to the trend observed in lycopene, the β‐Carotene content exhibited a significant reduction after drying (*p* < 0.05). While the fresh sample possessed the highest β‐Carotene value of 83.54 mg 100 g^−1^ DM, the values recorded in the dried products decreased to a range of 44.40–51.16 mg 100 g^−1^ DM. The highest retention among the dried samples was observed in the CO‐70°C group (51.16 mg 100 g^−1^ DM), whereas the lowest was found in the MW 240 W group (44.40 mg 100 g^−1^ DM). However, statistical analysis revealed no significant differences among the applied drying methods and conditions. As discussed in the color properties section, the primary mechanism driving the loss of lycopene and β‐carotene during thermal processing involves isomerization and subsequent oxidation reactions. While thermal treatment and exposure duration lead to degradation by disrupting the chemical structure of these bioactive compounds, the presence of atmospheric oxygen—particularly in CO—further accelerates this oxidative destruction. Petković et al. (2025) noted no considerable change in lycopene and β‐Carotene content in dried tomato pomace waste at 50°C and 70°C. Besides, Toledo et al. (2022) found no statistical difference in lycopene content of dried tomato skin at 55°C and 70°C. Surendar et al. (2018), Demiray et al. (2013) noted loss of lycopene and carotene in tomato powders and tomato slices after CO drying process, respectively. (Horuz et al. 2017b) similarly reported that MW drying caused lycopene loss in tomato slices.

### 3.7 5‐Hydroxymethylfurfural Content of Tomato Pomace Powder

Furan derivatives like 5‐hydroxymethylfurfural (HMF) are key intermediates of the Maillard reaction and caramelization (Meng et al. 2024; Akder et al. 2025), typically forming from hexose sugars under acidic conditions (Choudhary et al. 2021; Shapla et al. 2018). While HMF levels are regulated in Turkey (20–75 mg kg^−1^) due to potential toxicological concerns (Ünüvar 2018; Choudhary et al. 2021), the juice extraction process used to produce the TP in this study significantly reduced the soluble sugar precursors (glucose and fructose) typically found in tomatoes (Coklar and Akbulut 2010).

Consequently, HMF was not detected in fresh TP or any dried samples (Table 7). This absence could be primarily attributed to substrate limitation, as the removal of reducing sugars during juice separation prevented HMF accumulation despite the acidic environment (pH 4.69). These results align with Turgut et al. (2018), who reported no HMF in tomatoes dried at 60°C, and Li et al. (2022), who observed minimal formation even in sugar‐rich whole tomatoes. Thus, the mechanical separation of juice ensures the chemical safety of the final TPP regarding furanic compounds.

### 3.8 ATR‐FTIR Analysis of Tomato Pomace Powder

**FIGURE 6:**
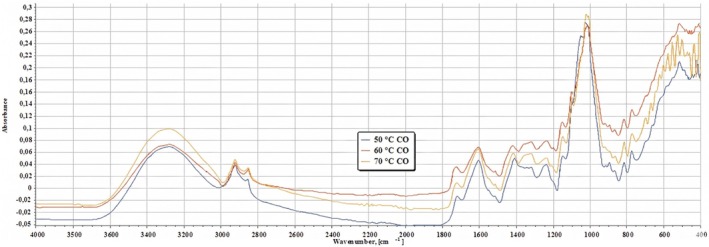
ATR‐FTIR spectra of CD dried tomato pomace.

ATR‐FTIR spectra of TPP dried at 50°C, 60°C, and 70°C (Figure 6) exhibited characteristic bands for polysaccharides (~3330 and 1200–900 cm^−1^), lipids (~2920, 2850, 1740 cm^−1^), and proteins (~1630–1650 cm^−1^). The absence of peak shifts confirms that drying temperatures within this range did not fundamentally alter the chemical backbone or cause significant degradation. However, a systematic increase in absorbance intensity was observed with higher temperatures (70°C > 60°C > 50°C). This phenomenon might be attributed to a potential physical measurement artifact related to surface contact quality between the sample and the ATR crystal. Lower temperatures (50°C) likely produced particles with a “gummy” texture and poor contact, whereas higher temperatures (70°C) induced “case hardening,” resulting in a harder, friable structure that fractured and packed more densely, thereby maximizing the absorbance signal.

**FIGURE 7:**
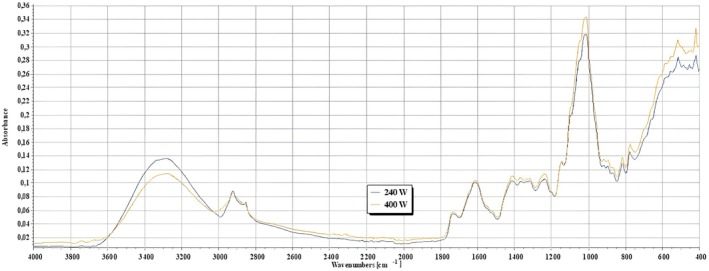
ATR‐FTIR spectra of MW dried tomato pomace.

Similarly, MW processing (240 and 400 W; Figure 7) maintained a consistent chemical profile. The significantly higher O‐H intensity (~3330 cm^−1^) at 240 W reflects higher residual moisture, while the 400 W sample exhibited stronger signals in the polysaccharide region (~1030 cm^−1^). This “relative concentration” effect occurs as the efficient removal of water at 400 W increases the proportional mass of heat‐stable components, such as cellulose and pectin, within the remaining solid matrix.

### 3.9 Principal Component Analysis of Tomato Pomace Powders

**FIGURE 8:**
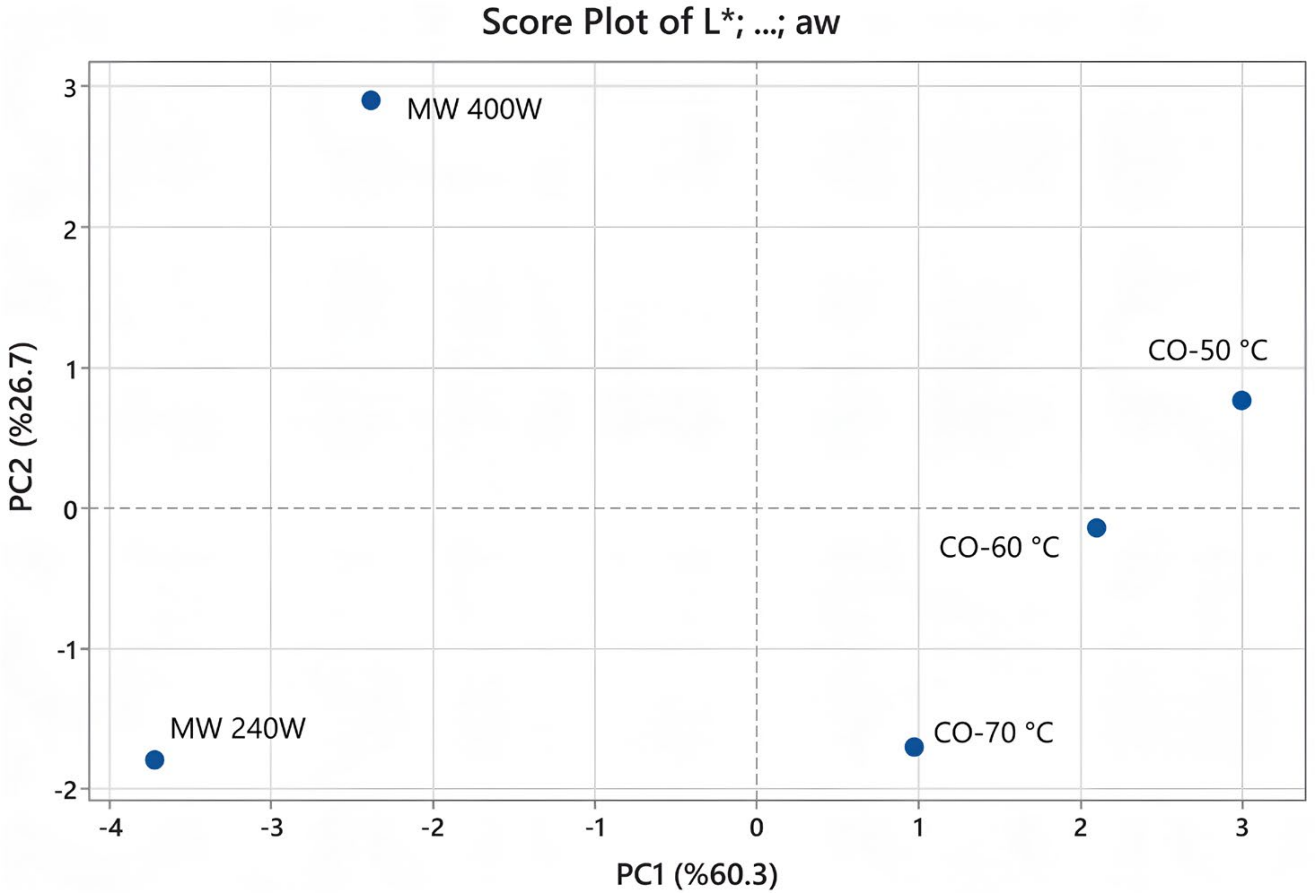
Principal component analysis of CO and MW dried tomato pomace.

Principal Component Analysis (PCA) was performed on 14 physicochemical parameters to visualize sample groupings and simplify the complex interrelationships between variables (Figure 8). The first two components accounted for 87.0% of the total variance (PC1: 60.3%, PC2: 26.7%), providing a robust two‐dimensional representation of the dataset. PC1 clearly discriminated drying methods: MW treatments clustered on the negative axis, associated with higher antioxidant capacity (AA: −0.320, AscA: −0.304, TPC: −0.269), while CO dried samples clustered on the positive axis, linked to physical stability (HR: 0.329, CI: 0.327) and carotenoid content (β‐Carotene: 0.286, lycopene: 0.285). This suggests a potential contrast where CO drying may favor physical and pigment stability, while MW drying might be superior for antioxidant retention.

PC2 further differentiated samples based on color (L*: −0.498, a*: −0.433) and TPC. Considering the specific objectives for cracker fortification imparting red‐yellow color, maximizing carotenoid contribution, and ensuring physical stability CO‐70°C emerged as the optimal candidate. Positioned in the positive PC1 and most negative PC2 regions, this sample mathematically represents the best balance of carotenoid retention and prominent red‐yellow pigmentation (a*: −0.433, b*: −0.197) compared to all other treatments. While MW‐400 W showed higher antioxidant potential, the necessary trade‐off favored CO‐70°C for its superior physical and chromatic suitability as a functional ingredient in bakery applications.

### 3.10 Color Properties of Crackers Fortified With Tomato Pomace Powder

**FIGURE 9:**
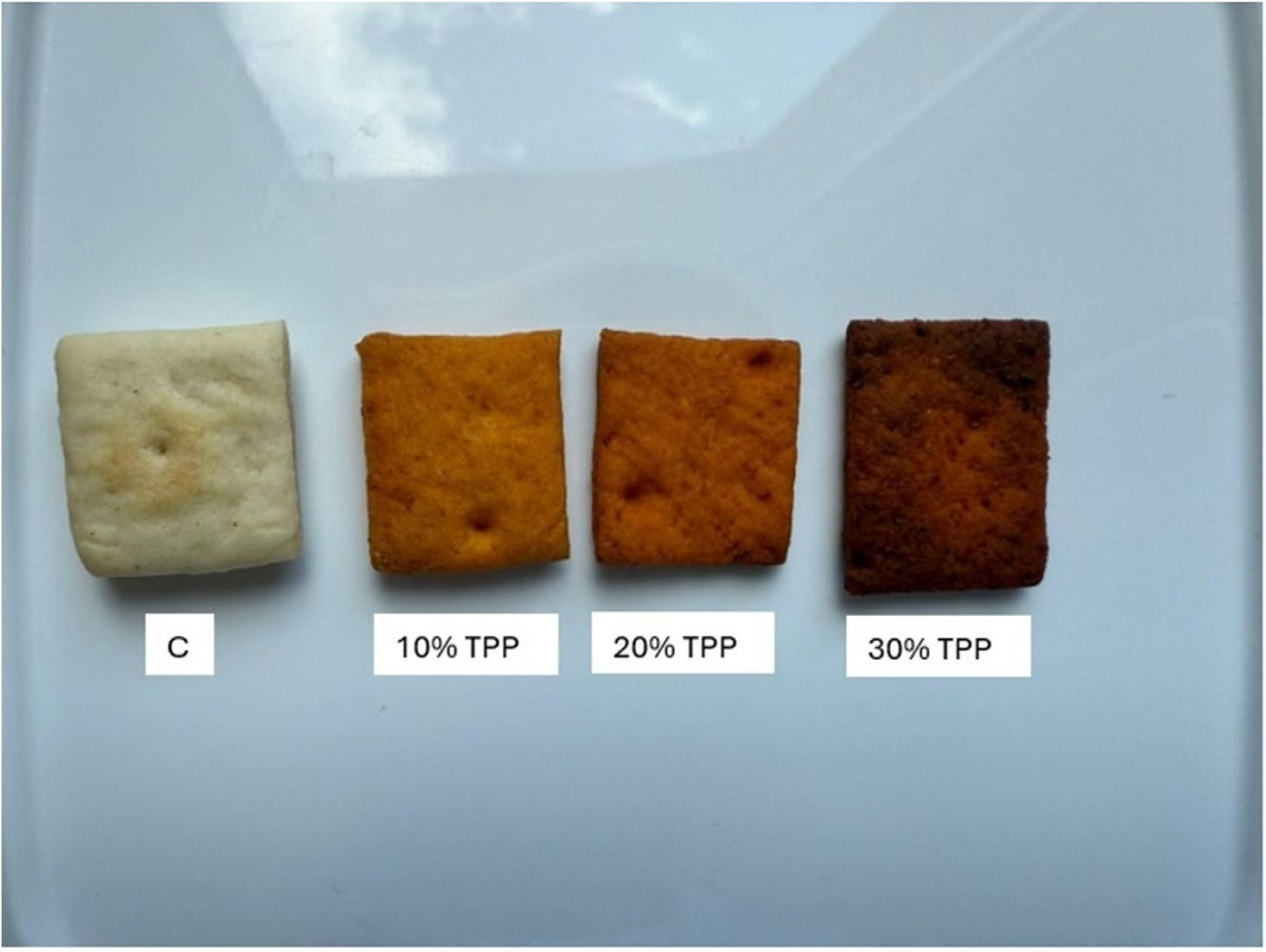
Crackers containing different ratios of tomato pomace powder.

Table 8 presents the colorimetric parameters of crackers fortified with varying concentrations of TPP. As anticipated, the incorporation of TPP significantly influenced the color characteristics of the final product (*p* < 0.05). A progressive decline in L* was observed with increasing TPP substitution levels; the L* value decreased from 74.48 in the control sample to 43.75 in the sample containing 30% TPP. This significant reduction in lightness is primarily attributed to the substitution of white wheat flour with TPP, which possesses a characteristic dark red pigmentation, as well as the intensification of non‐enzymatic browning reactions during the baking process. On the other hand, a* values exhibited a marked increase, rising from 11.46 in the control to values exceeding 36.09 in the fortified samples. This substantial elevation confirms the retention of lycopene pigments within the cracker matrix. Conversely, the yellowness b* values reached a maximum at the 10% and 20% substitution levels but significantly declined at the 30% level. This reduction suggests that at higher concentrations, the intense reddish‐brown color derived from both the pigments and the browning reactions masked the yellow coloration, thereby dominating the overall visual appearance. Isik and Topkaya (2016) reported similar L*, a* and b* trend crackers fortified with TPP. Figure 9 shows the crackers containing different ratios of TPP. Yagci et al. (2022) also noted that L*, a* and b* value decreased with the increment of TPP ratio in different flour and starch based extruded snacks.

**TABLE 8:** Color attributes of crackers fortified with tomato pomace powder.

Experiments	L* ± SD	a* ± SD	b* ± SD
Control	74.48 ± 2.41^a^	11.46 ± 1.29^c^	25.69 ± 2.18^c^
%10 TPP	56.13 ± 2.54^b^	30.15 ± 0.86^b^	54.06 ± 1.72^a^
%20 TPP	53.81 ± 0.90^b^	36.09 ± 0.90^a^	53.81 ± 0.92^a^
%30 TPP	43.75 ± 3.50^c^	33.99 ± 2.25^a^	39.83 ± 5.30^b^

*Note:* Different letters in the same column indicate significant differences with confidence of 95%.

Abbreviation: SD, standard deviation.

### 3.11 Bioactive Properties of Crackers

TPC, AA, AscA, lycopene and β‐Carotene contents of crackers which were produced in different formulations were given in Table 9. TPC and AA of crackers ranged from 19.75 to 138 mg GAE 100 g^−1^ DM and from 0.1 to 0.76 mmol TE 100 g^−1^ DM. AscA, lycopene and beta carotene contents of samples were found between 22.5 and 38.38 mg 100 g^−1^ DM, 0 and 130.42 mg 100 g^−1^ DM, 0 and 15.45 mg 100 g^−1^ DM, respectively. All bioactive properties of samples increased with increasing TPP. Especially, lycopene and beta carotene originated from TPP; control samples were not included in these compounds. Similarly, Isik and Topkaya (2016) reported that increasing tomato powder levels caused an increment in TPC and AA of crackers. Yagci et al. (2022) noted an increment in TPC, AA and lycopene content of different flour and starch based extruded snacks depending on the ratio of TPP. These increments can be explained by higher bioactive contents of TPP than wheat flour.

**TABLE 9:** Bioactive properties and water activities of crackers fortified with tomato pomace powder.

Experiments	TPC (mg GAE 100 g^−1^) ± SD	AA (mmol TE 100 g^−1^) ± SD	AscA (mg 100 g^−1^) ± SD	Lycopene (mg 100 g^−1^) ± SD	β‐Carotene (mg 100 g^−1^) ± SD	a_w_ ± SD
Control	19.75 ± 6.31^c^	0.10 ± 0.03^d^	22.50 ± 5.24^b^	ND	ND	0.37 ± 0.002^a^
%10 TPP	37.29 ± 6.59^c^	0.25 ± 0.03^c^	30.74 ± 5.10^ab^	37.44 ± 1.14^c^	4.02 ± 0.18^c^	0.31 ± 0.001^b^
%20 TPP	83.32 ± 11.11^b^	0.45 ± 0.03^b^	33.57 ± 6.32^ab^	105.46 ± 1.14^b^	12.03 ± 0.14^b^	0.27 ± 0.004^c^
%30 TPP	138.32 ± 14.05^a^	0.76 ± 0.06^a^	38.38 ± 8.44^a^	130.42 ± 0.51^a^	15.45 ± 0.18^a^	0.24 ± 0.001^d^

*Note:* Different letters in the same column indicate significant differences with confidence of 95%.

Abbreviation: SD, standard deviation.

### 3.12 ATR‐FTIR Analysis of Crackers

**FIGURE 10:**
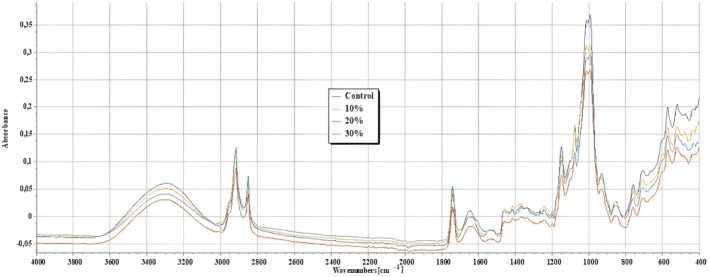
ATR‐FTIR spectra of crackers.

The ATR‐FTIR spectra of the control and TPP‐enriched crackers are comparatively illustrated in Figure 10. The control sample exhibited a typical starch‐dominated profile, with major bands at ~3330 cm^−1^ (O‐H stretching), ~2900 cm^−1^ (aliphatic C‐H), ~1640 cm^−1^ (bound water bending), and the characteristic glycosidic “fingerprint” region at ~1030 cm^−1^. With the incorporation of TPP, a new shoulder peak emerged at ~1740 cm^−1^, identifying the C=O stretching vibrations from pectin and lipids inherent in the pomace. This chemical signature confirms the successful integration of TPP into the wheat flour matrix.

A systematic decrease in the absorbance intensity of major polysaccharide peaks (~3330 and ~1030 cm^−1^) was observed as the TPP ratio increased (Control > 10% > 20% > 30%). This trend may be considered a physical artifact of the ATR technique rather than a chemical dilution. The fibrous and particulate nature of TPP increases the surface roughness and disrupts the homogeneity of the cracker matrix. This altered topography creates micro‐air gaps that weaken the contact between the ATR crystal and the sample surface, thereby reducing the effective sample volume analyzed.

These spectroscopic findings strongly corroborate the water activity (*a*_w_) results (Table 9), which decreased significantly with higher TPP substitution (*p* < 0.05). The high dietary fiber content—rich in cellulose, hemicellulose, and pectin—provides abundant hydroxyl groups that form strong hydrogen bonds with water molecules. By effectively binding “free water” within the matrix and providing a high specific surface area for moisture adsorption, TPP enhances both the shelf stability and the crispness of the crackers. Ultimately, the FTIR intensity trends indirectly reflect the physical impact of the increased fiber load, which is the primary mechanism behind the observed moisture‐binding capacity.

### 3.13 Sensory Analysis of Crackers

The sensory parameters of the crackers, including color, odor, flavor, hardness, crispness, and overall acceptance, are presented in Table 10. The 10% and 20% TPP formulations achieved the highest scores across all parameters, with no statistical difference between these two samples (*p* > 0.05). However, a significant decline in sensory acceptability was observed at the 30% TPP level. This reduction might be primarily attributed to the potential structural inhibition of the dough matrix; the excessive dietary fiber (cellulose and pectin) in TPP forms an overly rigid network that restricts dough expansion during baking, resulting in a dense and hard texture rather than the desired crispness. Furthermore, the increased surface roughness and micro‐voids identified in the FTIR analysis likely translated to a “gritty” mouthfeel at this inclusion rate. While 30% TPP maximized the bioactive contribution, the 20% formulation represents the optimal trade‐off, successfully balancing functional enrichment with the sensory and textural characteristics preferred by the panelists.

**TABLE 10:** Scores of sensory analyses of crackers fortified with tomato pomace powder.

Experiments	Color ± SD	Odor ± SD	Flavor ± SD	Hardness ± SD	Crispness ± SD	Overall Acceptance ± SD
Control	3.00 ± 1.29^b^	3.26 ± 1.05^a^	3.05 ± 0.78^b^	3.26 ± 1.19^ab^	3.42 ± 0.96^a^	3.16 ± 0.96^b^
%10 TPP	4.74 ± 0.45^a^	3.79 ± 0.85^a^	4.11 ± 0.88^a^	4.00 ± 0.94^a^	4.00 ± 1.00^a^	4.42 ± 0.77^a^
%20 TPP	4.32 ± 0.58^a^	3.79 ± 0.85^a^	4.00 ± 0.94^a^	3.47 ± 1.22^ab^	3.74 ± 0.99^a^	4.11 ± 0.88^a^
%30 TPP	2.42 ± 0.77^b^	3.11 ± 0.99^a^	2.84 ± 1.01^a^	2.95 ± 1.22^b^	3.47 ± 0.96^a^	2.89 ± 0.94^b^

*Note:* Different letters in the same column indicate significant differences with confidence of 95%.

Abbreviation: SD, standard deviation.

### 3.14 Principal Component Analysis of Crackers

**FIGURE 11:**
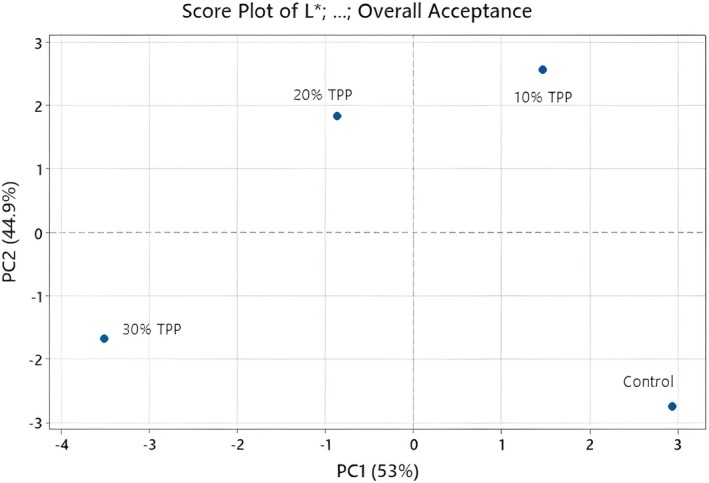
Principal component analysis of crackers.

PCA effectively mapped the critical differences between cracker formulations, with PC1 and PC2 explaining 97.9% of the total variance (Figure 11). PC1 (53%) functioned as the “Bioactive Enrichment and Color Axis,” where the negative direction (left) strongly correlated with TPP‐derived bioactives—including AA (−0.354), TPC (−0.353), lycopene (−0.346), β‐Carotene (−0.348), and AscA (−0.337)—alongside increased redness (a*: −0.274). Conversely, the positive PC1 region (right) was defined by the control cracker's characteristics, specifically higher brightness (L*: 0.325) and water activity (*a*_w_: 0.341).

PC2 (44.9%) emerged as the “Sensory Acceptability and Texture Axis,” where the positive direction (top) displayed a robust correlation with panelist‐preferred parameters: Flavor (0.367), Overall Acceptability (0.365), Odor (0.359), and Crispness (0.367). Mapping these axes revealed that while the 30% TPP sample maximized bioactive contribution, it fell into the negative PC2 region due to reduced sensory scores. The 10% TPP sample, although sensorially successful, remained weak in bioactive density.

Ultimately, the 20% TPP formulation stood out as the optimum choice. Positioned in the upper‐left quadrant (negative PC1, positive PC2), it successfully combined significant bioactive enrichment with high sensory acceptance. This strategic position mathematically confirms that 20% substitution represents the “sweet spot,” providing a functional upgrade without compromising the palate‐pleasing characteristics—flavor, crispness, and color—desired by the panelists.

## 4 Conclusions

This study demonstrates that TP can be effectively upcycled into a functional food ingredient, directly supporting circular economy and zero‐waste initiatives. The investigation of drying kinetics revealed that moisture removal is primarily governed by external convective resistance, identifying surface evaporation as the rate‐limiting step. While conventional models provided satisfactory predictions, the ANN approach offered superior robustness and flexibility, proving to be a highly effective tool for real‐time process control in industrial drying.

A strategic trade‐off was identified regarding the drying method: while microwave drying at 400 W maximized energy efficiency and antioxidant retention, CO drying at 70°C was found to be the most suitable for bakery applications due to its superior preservation of lycopene, β‐carotene, and characteristic red pigmentation. The chemical safety of the produced powders was confirmed by the complete absence of HMF across all treatments.

Incorporating TPP into crackers significantly enhanced their nutritional profile, transforming a standard snack into a source of dietary fiber and essential pigments. ATR‐FTIR analysis suggested that the high fiber load may have physically modified the matrix, effectively binding free water and enhancing shelf stability. While higher substitution levels maximized bioactivity, the 20% TPP formulation emerged as the optimal “sweet spot,” providing substantial functional benefits without compromising the sensory acceptance, crispness, and flavor profiles preferred by panelists. These findings suggest that TPP is a viable, sustainable, and health‐promoting additive for the development of value‐added snack products.

## Author Contributions

**Fadime Begüm Tepe:** methodology, conceptualization, investigation, data curation, writing – original draft, writing – review and editing. **Tolga Kağan Tepe:** methodology, conceptualization, investigation, project administration, data curation, formal analysis, writing – original draft, writing – review and editing.

## Funding

This research was supported by Giresun University (Grant number: FEN‐BAP‐A‐110225‐26). In addition, open access funding provided by the Scientific and Technological Research Council of Türkiye (TÜBİTAK).

## Ethics Statement

This research was approved by Giresun University, Social Sciences, Natural Sciences, and Engineering Research Ethics Committee (Date: 07.05.2025 and Number: 05/300) in terms of sensory evaluation.

## Conflicts of Interest

The authors declare no conflicts of interest.

## Data Availability

The datasets generated during and/or analyzed during the current study are available from the corresponding author on reasonable request.
